# Practical Observations on the Principal Diseases Affecting the Health of European and Native Soldiers in the North-Western Provinces of India, &c.

**Published:** 1846-04

**Authors:** 


Practical Observations on the Principal Diseases affecting the Health of European and Native Soldiers in the North-Western Provinces of India, &c. By *W. L. Macgregor*, M.D., Surgeon, 1st European Light Infantry. 8vo. pp. 316. Calcutta. Thacker and Co.

We introduced Dr. Macgregor to the acquaintance of our readers, in our Number for last July, as the principal writer in the new East India periodical, entitled the Quarterly Medical and Surgical Journal for the N. Western Provinces; and we then gave a variety of extracts from it, to point out the line of treatment which, according to his statement, has proved so successful in a variety of the diseases of India, more especially in Bilious Remittent Fever, Epidemic Cholera, and Dysentery. The present work of our author contains numerous illustrations of the same subject; and, as his practice—which is in some respects original—seems unquestionably to have been of decided advantage in many (we do not say all) cases, it may not be without profit that we briefly recal the attention of our readers to its leading features.

Dr. M. most emphatically, and over and over again, asserts that a uniform and indeed an essential feature and pathological character (as ascertained by dissection in fatal cases) of Fever—including the intermittent and remittent forms—epidemic Cholera and Dysentery, is a distended and obstructed state of the gall-bladder; and, basing his therapeutic views upon this foundation, he with great earnestness maintains that the leading object in their treatment is to unlock the pent-up bile, and to eliminate and discharge it from the body.* He states, and with truth certainly, that the

---

* Dr. Mouat, whose recent pamphlet we have noticed in a subsequent part of the present Number, observes that, "it is well known to all who have had a practical acquaintance with this fearful pestilence, that not an atom of bile is visible in the *ejecta* of a true Cholera case. The gall-bladder in fatal instances is usually found distended with bile, and the patient may in general be considered as doing well, when the character of the excretions becomes changed."

alarming symptoms in each of these epidemic diseases never subside until there is the appearance of bile in the stools ; and that, as long as the alvine evacuations consist of congee-like water, slime, or mucus, we cannot with propriety recognise a decided amendment in any case, however much the violence of the other symptoms may have abated. The action of Vomiting, that so generally occurs in the early stage of the morbid process, is to be regarded as an effort of Nature to effect the dislodgment of the obstructed bile : great caution should therefore be used in hastily checking this salutary *nisus* of the system. Now, this is a most important practical point, and one that requires to be well considered by every medical man in the treatment of all pestilential or malignant epidemic diseases. A vast deal of harm has been done by giving large doses of opium, or by the use of other sedative means—at the commencement not only of spasmodic cholera, but of various fevers, European as well as tropical—with the view of checking the severe vomiting that is often present at first. This is unquestionably a most erroneous and hurtful practice. Indeed, it may be laid down as a universal rule, in the management of all pyrexial diseases without exception—and Cholera, we may remark, is regarded by our author, as it has been by many preceding writers, as a concentrated and overpowering form of Malarious Fever—that spontaneous vomiting, at the onset of a febrile attack, should be promoted for a time, and seldom or never suddenly arrested. With this view, the patient should be encouraged to drink two or three tumbler-fuls of hot water, to which some common salt or sal volatile has been added; and, should these means not succeed in bringing on the rejection of some bilious matters from the stomach, it will very generally be prudent to administer a full dose of Ipecacuan without delay. Sydenham and all the old authors of repute give this advice ; it is one of sound practical value.

It is well known, too, that some of the best writers on India Cholera have strongly recommended the use of powerful emetics in the treatment of this formidable pestilence; and we can bear witness, from the results of our own experience, of the propriety of the practice. Dr. Macgregor is of the same opinion. The very large dose of Croton oil, which he usually exhibited, viz. five drops, had generally the effect of at first increasing the vomiting ; afterwards, it appeared to quiet and subdue the excessive irritability of the stomach. This might be owing to the opium given at the same time; but we may take this opportunity of stating, that by far the best means to allay spontaneous vomiting, when it occurs in the course of various diseases, is to make a strong impression upon the stomach, by giving a full dose of Ipecacuan. Cases are continually occurring to us where this practice has succeeded, when every means to check the symptom by effervescing draughts, opium and so forth, had entirely failed. We might add that, in many cases of Diarrhœa, the action of a decided purgative will often serve to arrest the intestinal discharge more effectually, as well as more safely, than any astringent.

That Dr. Macgregor has greatly exaggerated the value of his favourite remedy—large doses of Croton oil, alone or combined with Opium or Henbane—in the treatment of East India Cholera, we have no doubt. Such extraordinary efficacy as he claims for it—on more than one occasion he tells us that it has never failed—at once begets a suspicion as to either the candour or the experience of the writer. But, making allowance for a considerable amount of extravagance in his praise, we have no hesitation in saying that we should certainly be inclined to give a fair trial to the practice which he so earnestly recommends. He is favourable to the employment of venesection in the very early stage of the disease, before the characteristic symptoms of prostration are fully developed; but often, we need hardly say, there is no time or opportunity afforded for the employment of this remedy. With respect to the use of direct emetics, we find the following observations:—

"Emetics, particularly Tartar Emetic and Ipecacuanha, have been extolled; and certainly the former medicine has been found, in many instances, capable of removing cholera, when free vomiting could be induced by persevering in its use. Ipecacuanha, in sporadic cases, is often useful in arresting the disease.

"The effect of emetics both in cholera and bilious remittent fever is the same, namely, that of giving a shock to the system, whereby the gall-bladder is occasionally emptied, which is the first step in the cure of both diseases, by whatever means accomplished." P. 67.

But his chief reliance is upon Croton oil and Opium in large and repeated doses. As we have already described the mode of exhibiting these medicines in our notice of Dr. M.'s writings, in the new East India Journal, it is unnecessary to enter into particulars at present. We shall only quote the following remarks as to the action or physiological effects of Croton oil when given in large doses.

"Many vegetable purgatives act as sedatives in large doses, while in smaller ones they are irritants; this is particularly observable with regard to Croton oil, a substance usually given in the dose of half a drop, or a minim or two at most; in such doses it acts as a drastic cathartic, causing great uneasiness, and often nausea and vomiting; if the same medicine be administered in the dose of five drops, it acts as a direct sedative, and a powerful purgative; and as such is an invaluable medicine in the treatment of diseases of warm climates, particularly biliary ones. Another medicine of great powers as a sedative, tartar emetic, may be adduced; its wonderful effect, as a sedative in diseases of the chest, and affections of the brain depending on increased irritation, or even inflammation, has been long acknowledged; and in Cholera its use has often been marked, and successful when persevered in until free vomiting has taken place." P. 13.

We must not forget to mention that our author is a decided non-contagionist as regards the Epidemic Cholera of India. His name must therefore be added to the list of those gentlemen whose views upon this question Dr. Copland, in the last number of his Dictionary, strives to shew are utterly untenable. For our own part, we must confess that we are by no means satisfied with the accuracy of the doctor's elaborate reasonings upon not only this, but upon some other points in the history of the Cholera pestilence. We may briefly allude to one of these. Dr. C. argues that it is a disease essentially *sui generis*, and that it is quite distinct from the sporadic malignant Cholera, such as was wont to be met with in India before the outbreak of the great epidemic of 1817, at Jessore in the delta of the Ganges, and which is never altogether absent in some part or another of the peninsula.

We see no good reason for joining with him in this respect; nor can we admit the essential difference of diseases which may exhibit exactly the same phenomena alike during life and after death, merely because the one may prevail and spread epidemically, and the other occurs sporadically, or within a very limited district. That the symptoms of the two diseases are nearly the same may be fairly inferred from Dr. Copland's own definition of spasmodic Cholera—the Mort de Chien of French writers. Here they are—*Vomiting and purging of watery matters, without any appearance of bile; spasms, violent and extending generally through the frame; speedily followed by sinking of the powers of life.* When, in addition to these features, we are told that, in some severe cases, the pulse can scarcely be felt within four hours from the attack, and that patients often die in the space of ten or twelve hours, it seems scarcely reasonable to maintain that the disease is essentially different from that epidemic form of which we have heard so much during the last 25 years, and which visited our own shores in 1831-2. Besides the argument of the close resemblance in the phenomena of the alleged two diseases, it deserves notice that most East India practitioners certainly did not regard the pestilence of 1817 to be a *new* disease, as Dr. Copland all along maintains. Our limits will not, as a matter of course, permit us to discuss this question at present. So strongly convinced, however, is our learned opponent of the truth of his opinions upon this point, that he wishes (vide our last Number, page 147) to abolish the appellation of Cholera, as applied to the epidemic or pestilential form, altogether, and to substitute the somewhat strange one of "Asphyxia pestilenta" (pestilens or pestifera?)* Medical men are seldom fortunate in their coinage of new names; and certainly the present instance is one of the least successful. That proposed a few years ago, in the Madras Journal, by Dr. Murray, "Adynamia algida," or "Acholera adynamica," is not much better. Why not Pestilential Cholera?—especially as we have seen that, although the generic name may, strictly speaking, denote a profluvium of the bile, there is admitted to be, in one of Dr. Copland's own species, "vomiting and purging of watery matters, *without any appearance of bile.*"†

From Cholera we pass on to notice briefly the treatment recommended by Dr. Macgregor in the Remittent Fever of India. The following extract presents a summary of his views:—

"When first seen, and the earlier the better, give the emetic draught, or when high arterial action is present in a young subject, bleed according to the age and effect produced: then give the croton pills, and watch the result; if Apyrexia be produced and no heat of scalp remain, give the quinine in a ten-grain dose. If the tongue be foul and loaded, exhibit the senna and salts, and for the heat of scalp apply leeches: should these fail, the probability is, that the essential cause is not removed, and the croton oil is to be repeated, and united in such cases

---

* This is the first of the pestilences described by Dr. C.; the other two being Yellow Fever, and the Plague. The former of these receives the appellation of *Pestilentia hæma-gastrica;* a curious circumstance certainly, when we consider that its specific distinction points to two of the most characteristic features of Epidemic Cholera—viz: the state of the blood, and that of the abdominal viscera.

† It may be worthy of notice that the Greek word χολερα signifies a water-pipe or gutter to carry off water, as well as a disease accompanied with a discharge (ρεω) of the bile (χολη).

with not less than five grains of extract of henbane. The second dose will generally remove the essential cause, but if the brain be involved, the head must be shaved, and the leeches repeated, the number varying from eight to twenty; purgative enemata containing Ol. Tereb. are to be injected for emptying the rectum in very severe cases; and where a little heat of scalp remains the quinine may be given, but combined with the croton oil, which often, under these circumstances, produces free vomiting of bilious matter from the stomach." P. 253.

In a subsequent part of his work, our author insists more earnestly upon the necessity of the free use of the lancet whenever there is pain or decided uneasiness in the region of the liver, or when the scalp is hot, and the cerebral symptoms indicate arterial over-action or venous congestion of the encephalon. After emphatically reminding the reader that he only uses Croton oil "as a means of removing the essential cause (the distended and obstructed state of the gall-bladder), and that other means must be employed for the effects produced by the action of that cause on the system," he goes on to lay down certain rules for the administration of Quinine:

"No matter what the state of the tongue may be, or whether the patient has a bilious look, or even if the bowels be not moved freely, provided a complete Apyrexia be present, the quinine must be given in a large dose, or, if the time will permit, a purgative draught may be given, followed in one, two, or three hours by the quinine. This injunction applies more particularly to severe Bilious Fever, in which an exacerbation is liable to be followed by fatal results. And as the exacerbation occurs most frequently as the heat of the day advances, the purgative, when indicated, is to be given early, and quinine to the extent of ten grains before 8 A.M." P. 253.

"But when the Apyrexia is not complete, as indicated by the heat of scalp, and state of the pulse and skin, but particularly the first, quinine is not to be administered, unless in some very severe cases, where death by effusion is sure to follow an exacerbation; and here the croton oil must be united with the quinine, in the proportion of five drops of the former to ten grains of the latter; but even here the case must be watched, and if the cerebral symptoms be increased, no time is to be lost in removing them by leeches, shaving the head, cold, particularly ice and blisters; while purgative enemata are thrown up: this is a case which will require great attention, and the medical officer should never leave his patient, until the danger is over." P. 252.

As a specimen of Dr. Macgregor's practice, we shall give one of his cases reported in the Appendix.

"Gunner Patrick Kassick, 2nd Troop, 1st Brigade, Horse Artillery, æt. 23. Admitted 12th July, 1842, with head-ache, and other febrile symptoms.

Mittatur sanguis ad lb. j.
Sumat Haust. Emet.

"13th. Feels better: slight head-ache: skin moist: pulse natural: tongue loaded.

Sumat Ol. Croton, gtt. v.

Ext. Hyosc. gr. ¼.

"P. M. Has head-ache, and there is great heat of scalp: pulse full and hard: bowels open.
Repet. Venæsectio, et si dolor adsit
Abradatur Capillitium et
Applic. Lotio frigida,
Injiciat Enema Domest. post tres horas.

"14th. There is some heat of head, and slight pain: feels better: some acceleration of the pulse, and increase of temperature: tongue whitish, and loaded in the centre. Applic. Hirud. xij. temporibus;
et habeat Pil. Croton. C.

"P. M. Is now bathed in perspiration, but was very feverish and restless during the day: the medicine produced several stools: has no head-ache now, nor local pain. ℞. Aq. Acet. Ammoniæ ℥iv.
Aquæ ℥viij. M.
Sumat ℥j. omni horâ.

"15th. Is now perfectly cool.
Sumat Sulph. Quininæ gr. x.
et habeat Mist. Quin.

"P. M. Has not had an exacerbation of fever, but complains of headache though the scalp is tolerably cool.
Applic. Hirud. xij. temporibus.
Repet. Pil. Croton C.

"16th. Bowels freely moved. Cont. Mist. Sulph. Quininæ.

"17th. Complains of great weakness: no febrile symptoms.
Cont. Mist. Quininæ.

"18th. Appetite improving; complains only of debility. Cont.

"20th. Discharged.

"N. B. The lancet was employed on account of the head symptoms, which it subdued, but the fever yielded to the Croton Oil." P. vii.

We may here remark, that the croton oil in large doses has been used with great advantage in the remittent fever of the West Indies: it was first recommended, we believe, by Dr. Tegart, Deputy Inspector of Hospitals, about 18 or 20 years ago. Dr. Macgregor was probably not aware of the circumstance.

The following extract from a letter of Dr. Hacket—in the number of the Medico-Chirurgical Review for Oct. 1832—will probably be read with interest by him as well as by other readers. After having alluded to the "astonishing and wonder-working effects" of the croton oil in his own experience, Dr. H. quotes this passage from a communication addressed to him by surgeon Cunningham.

"Five cases of remittent fever were treated in the Regimental Hospital during the quarter. Two cases were also treated in quarters, of peculiar severity, viz. one officer, Major Gibson, of the 86th—the other, the wife of the Serjeant Major. These were, in particular, aggravated cases, attended with intense degree of gastric derangement at the commencement of the attack. In the whole of these cases, the beneficial effects of the croton oil, in suddenly and effectually arresting the violent irritability of stomach, and in alleviating the urgent febrile symptoms, were particularly apparent; the quantity administered was *four drops* for the first dose, assisted by active purgative enemata and warm bathing, or the cold shower bath—the latter was found the most beneficial: a second dose of the oil, to the extent of one-third to *two drops* was in some of the cases found necessary. The sulphate of quinine, in doses of from 10 to 12 grains daily, was had recourse to at as early a period as circumstances would permit. Venesection was in none of the cases had recourse to; no instance of protracted convalescence occurred. There is every reason to conclude that, had the excessive irritability of stomach not been arrested by the use of the croton oil, and had the symptoms been combated, as formerly, by the use of other remedies, black vomit, with exhaustion, must have soon supervened, with a fatal termination."

It appears, therefore, that the bold practice of our author has been, in some measure, anticipated.

Our readers may naturally wish to know Dr. Macgregor's opinions as to the effects of the liberal administration of Calomel in the treatment of the fevers of the East Indies. On the whole, he appears to be not very favourable to the employment of this, unquestionably much misused and often dangerous—when indiscriminately or immoderately given—remedy. His words are:—

"Calomel is not a medicine to be lightly dealt with, and its power, both as a sedative and purgative, there is no man more inclined to concede than myself. I had used it for years in the cure of Fever and Dysentery, and in the latter with great success; but I could not be blinded to its pernicious effects on the constitution; and when, in addition to these, I found that the drug failed to cure severe cases of Bilious Fevers, while from some idiosyncracy, it aggravated certain cases of Dysentery, I was obliged to view it with suspicion, and endeavour to find substitutes which were not only equal, if not superior, to calomel in curing these complaints, but which possessed the advantage of not destroying the constitution, or inducing such serious disorders as Dry Gangrene, Ulceration of the Intestines, and incurable Dropsies.

"It is only when we trace the pernicious effects of calomel from first to last, that we are horrified at the uncertainty of such a powerful remedy." P. 235.

With respect to Intermittent Fever, we do not find much in Dr. Macgregor's work, that calls for notice. His explanation of the cause of the rigors in the first stage is ingenious, if not satisfactory.

"When speaking of hepatic complaints, I have observed, that rigors are often witnessed in cases where the gall-bladder has been impacted with diseased bile for years; and this circumstance leads me to infer, that the stage of rigor in Intermittent Fever is the effort of Nature to expel the contents of the gall-bladder, and the succeeding hot stage is the really diseased action, which, when terminating in the sweating, is removed, whereas, when the rigors are slight, or do not occur, as is often witnessed in Remittent Fevers, the efforts of Nature are ineffectual in throwing off the disease, and a distinct intermission does not occur, but only a partial remission: experience would seem to confirm this view of the subject, both with regard to the effects and the cure of Fever." P. 247.

With respect to treatment, it is summed up in a few words: "the essential cause is to be removed by the Croton oil, and the accession of the intermittent, and the exacerbation of the remittent, paroxysm prevented by Quinine; the effects produced on the system being combated by the lancet, leeches, blisters, cold, and other antiphlogistic measures."

*Dysentery.*—In this disease our author most energetically contends that there is invariably the same morbid condition of the biliary organs present, as exists in Cholera and Fever; and that the treatment of it, although requiring considerable modification in some respects from the highly inflammatory and irritated condition of the bowels, should be conducted upon the same principles.

"I had assumed," says he, in the latter part of his work, "as the essential cause of Dysentery, the distended gall-bladder, and the absence, in consequence, of cystic bile in the colon: nothing has occurred to shake this opinion, for in every fatal case of the disease, the gall-bladder has been filled with dark viscid, or ropy bile; or, if the bile appeared in the alvine evacuations, it was a thin greenish fluid, being in fact, the hepatic bile not subjected to the peculiar action of the gall-bladder. Such a fluid does not appear capable of producing the soothing, healing action of cystic bile." P. 265.

And again:—

"From the absence of the bile in the colon, its contents become acrid; the mucous coat is irritated, and the bowels, instead of propelling the fæcal matter, puts on a diseased action called Dysentery, as evinced by griping, straining and frequent alvine dejections of thin whitish matter; the same tinged with blood, or the latter mixed with mucus and slime; all these appearances may occur in the first or irritative stage, and still no inflammation be present; if these slight symptoms be overlooked, more particularly what is called Diarrhœa, or thin whitish stools, the irritation passes to the stage of inflammation, and this often speedily, in the case of Europeans; even here the disease is under control by means of the lancet, sedatives, and counter-irritants; if not checked, ulceration succeeds inflammation, and sooner or later involves the whole gut from the cœcum to the rectum, terminating in death." P. 263.

According to Dr. M's observation, Dysentery may be said to arise from the same (or a very similar) malarious poison or miasm which produces Remittent Fever. The two diseases often appear at the same time; while, in other instances, they alternate with, and succeed each other. "Cholera, Fever, and Dysentery are but links of one chain." The great tendency, however, to inflammation in the last disease demands the more prompt and free use of the lancet than in the other two; but the practitioner must never forget, in his antiphlogistic treatment, the paramount importance of evacuating the gall-bladder and promoting the discharge of the bile into the bowels. An emetic should be given at first; this may be followed by a dose of Castor oil combined with tincture of Henbane. After the bowels have been unloaded, an opium pill, either alone or united with quinine, should be administered. If we have reason to believe that the obstructed state of the gall-bladder continues, five drops of Croton oil with five grains of extract of Henbane will be found useful. In acute dysentery, large doses of Calomel, in combination with Opium, are unquestionably often of great efficacy in subduing the violent irritation of the bowels; but, in the chronic form of the disease, this medicine is decidedly injurious, inducing rapid emaciation, and, in too many cases, fatal consequences. For the last six or seven years, Dr. M. seems to have discontinued the use of calomel nearly altogether in the treatment even of acute Dysentery; and we believe that we are not far from the truth in saying that our medical officers, in the present day, do not order a tithe of the quantity of this often-abused medicine in the diseases of India, that was generally given twenty years ago.

To illustrate our author's usual treatment of acute Dysentery, we select the following brief report of one of his cases.

"Corporal John Woods, No. 3rd Company, 1st European Light Infantry. Æt. 34. Admitted 16th July, 1843.

"July 16th. Since yesterday, he has had dysenteric symptoms, stools frequent, bloody and mucous, attended by great tenesmus; on firm pressure, there is pain felt in the ascending colon and also in the transverse, but none in the cœcum nor in the descending portion: tongue red: no white crust or even a white spot.

Mittatur Sanguis.
Sumat postea Haust. Sedativ.

"17th. The bleeding removed the pain, but, in taking the draught, he was seized with severe spasms and rigidity of the muscles: vomited and the bowels were freely moved: stools fæcal, light-coloured: he took the Pil. Croton c̄ Opio last night, and had some straining with the evacuations which consisted of serous matter, without blood or mucus.

Repet. Pil. Croton. gtt. v. Opii gr. iij.

" 18th. Has no griping or tenesmus.

Sumat Opii gr. iij.

Sulph. Quin. gr. v.

" 19th. Bowels quite regular.

Nulla Med.

" 20th. Discharged." P. cvii.

We now proceed to notice some of the other contents of his volume.

*Functional Diseases of the Liver.*—The following is the description which our author gives of Congestion of this organ.

" The individual has a sense of weight in the right side, sometimes increased on full inspiration, but seldom on pressure; there is not actually pain, but a feeling as if some enlargement had taken place; the sensation is generally confined to the region of the liver, extending from the scrobiculis cordis towards the spine: when this condition of the liver has existed long, a yellow tinge of skin is not an uncommon occurrence, so that the individual is termed bilious; he becomes weak, and dislikes any exercise, either of body or mind, occasionally he feels chilly, at other times heated; with a dry skin, and the secretions become scanty, particularly the saliva and urine. The mouth is parched, and the phlegm becomes thick and inspissated: in the aggravated form, there is difficulty in lying on the left side; and in severe cases, the individual lies either on his back or right side; when attempting to turn from the latter position, he feels a weight in his right side, and is often led to suppose that the liver is diseased; that its volume is increased in bulk, there can be little doubt, from the additional quantity of blood in the viscus." P. 125.

The patient becomes more and more distressed with distinct rigors, usually accompanied with much disorder of the stomach, and with great uneasiness in the region of the liver: the occurrence of these rigors is apt to alarm the physician and make him suspect impending suppuration. They often indicate an effort of Nature to remove the dark inspissated bile from the gall-bladder; " for in old cases," says Dr. M. " I have invariably witnessed a severe rigor of this kind to be followed by the dejection of dark bile, more especially if any purgative has been administered." The bowels are very generally sluggish, and the evacuations are never quite healthy; they are either very dark, perhaps almost black, or they are clay-coloured. The head almost always suffers more or less severely; excessive drowsiness is a very common symptom. At other times, the patient can get no sleep; he is restless, and irritable; his temper becomes peevish, and his mind incapable of application to any subject. Congestion of the liver is an exceedingly common malady among our soldiers, European as well as native; and one cause of the great mortality of Remittent Fever, Cholera, and Dysentery among the East India troops is unquestionably the circumstance, that so many patients are affected with hepatic congestion at the very time when they are seized. At a subsequent part of his work, Dr. M. remarks that " seldom or ever do we meet with sporadic cases of these diseases, where a costive state of the bowels has not existed for some time."

In the *treatment* of Congestion of the Liver, our author gives a decided preference to Croton oil in powerful doses over Calomel or any other mercurial preparation. He is of opinion that much harm has been, and still is, done by the indiscriminate use of large doses of calomel in this and other diseases of the liver, which are not truly and distinctly of an inflammatory nature. The malady, for which it was given, may have indeed been cured; but the constitution in too many cases remains very seriously injured. There is no such objection to Croton oil, even when its effects are violent. The usual dose is five drops, combined either with extract of Henbane or with Opium. The dose requires to be repeated every alternate night, until the gall-bladder is thoroughly emptied, and the stools have lost that dark gelatinous appearance—which perhaps they have had for weeks or even months—and acquired the characters of healthy excretion. Another potent medicine, which Dr. Macgregor appreciates very highly, is tartrate of Antimony. His practice has at least the merit of boldness and decision; his prescriptions are certainly not mere *placebos*.

"The use of Tartar Emetic in congestion of the liver may be required, and if so, the following pills will be found powerful ones in producing a sedative, purgative, and diaphoretic effect:

℞. Ol. Croton gtt. v.

Tart. Antim.

Opii, āā gr. iij. M.

Divide in pil. ij. h. s. s.

"When necessary, the quantities of opium and tartar emetic may be doubled, and pills of this strength are often retained when the former are rejected. Where these medicines are given in functional disorders of the liver, they often produce a sensation as if something were working its way from under the margins of the right false ribs, and both there, and in the left hypochondriac region, actual pain is sometimes induced followed by an alvine evacuation, the nature of which varies according to the stage of the disease. In old obstinate cases, the operation of these medicines produces nothing but light-coloured, or whitish dejections, and affords no relief to the weight in the sides indicative of congestion and distended gall-bladder: great irritation of the stomach, amounting often to rejection of its contents may, likewise, follow each exhibition of the medicine; nothing, therefore, but the most determined perseverance on the part of the patient and practitioner can remove the disorder; and until the bile appears in the stools, either in a diseased or healthy state, little or no progress is made in the cure of the complaint." P. 135.

The warm bath is always a useful adjuvant in the treatment of Congestion of the Liver. After recovery from a severe or protracted attack of this disease, the patient will generally require to have a change of climate. A sea-voyage is one of the best restoratives. Before quitting the subject of Hepatic Congestion, we are tempted to give the following extract, on the *state of the tongue* as affording valuable diagnostic signs in biliary complaints.

"The state of the tongue is a most important subject in the treatment of biliary complaints. In congestion of the liver the tongue is generally whitish, and sometimes blanched, but not loaded; while, in inflammation of serous or mucous membranes, it is for the most part clean, red, and glistening; as congestion ceases, the tongue becomes loaded and moist; in forming a diagnosis, therefore, between congestion and hepatitis, the state of the tongue may, in some measure, assist us: for a red, clean, glistening tongue indicates the want of secretion, if not the presence of inflammation; while the white blanched tongue attends the action of secretion of bile, though the latter may be absent from the bowels, and lodged, either in the gall-bladder or stomach; when the tongue is quite white it evinces great irritation of the mucous membrane of the intestines from want of the presence of bile, and this tongue is witnessed in the worst forms of Bilious Remittent Fever. In recovery from congestion, the tongue becomes loaded, and begins to assume a natural clean appearance when the bile has been fairly restored to the bowels, beginning first at the tip and edges; so long as the tongue remains loaded and moist, the use of purgatives is indicated; and the infusions of Senna and Chirayita may be administered in combination with saline medicines." P. 136.

In severe and obstinate *Jaundice*, Dr. Macgregor has recourse to his favourite chologogue pills, combined with an unusually large dose of the antimonial. The formula he gives is this:—

℞. Tart. Antimonii gr. vj.
Opii (pill) gr. iij.
Olei Crotonis ♏v.

to be made into two pills with crumbs of bread:—"the tartar emetic acts here as a powerful sedative and antispasmodic, without necessarily producing in such a dose either nausea or vomiting."

Dr. M. thinks favourably of the Nitro-Muriatic Acid Solution, used either as a general bath, or applied as a wash to the surface. The internal use of the Nitric acid may also be often had recourse to with advantage.

Even for the relief of mere habitual *Constipation*, not to mention positive obstruction of the bowels, our author recommends his panacea of Croton oil (five drops), combined with Opium or Henbane; advancing the following very questionable reason for this preference:—"Instead of being obliged to repeat this medicine every other day, an interval of a week, or even a month, may elapse without any necessity for taking medicine; surely it is better thus to undergo the inconvenience of taking two small pills once a week, or once a month, than keep swallowing, or feeding on, pills daily."

We need scarcely caution our readers against such a line of practice. He goes on to say, that "no one need be alarmed at the apparently large dose of croton oil, a medicine supposed to act efficiently in a one-drop dose, or even half that quantity, for the same holds good of other powerful purgatives and sedatives. It is well known that a few grains of calomel act as a stimulant and purgative; while a scruple, or even half a drachm, has the opposite effect, or that of a sedative and astringent: in opium we find a medicine which, given in a grain dose or less, acts as a stimulant; while exhibited in doses of three or more grains it has a direct sedative effect." There is much fallacy, if not positive error, in this reasoning; the data, on which it is based, we are by no means inclined to admit.

The admirer of the Virginian weed may appeal to Dr. Macgregor as an authority in favour of its use:—

"As a means of relaxing the bowels, the use of tobacco may be mentioned; this article is extensively employed in the form of cheroots, the smoke of which inhaled into the mouth and palate appears to assist the action of the bowels, and does not impair digestion, if the mouth be not deprived of saliva by the practice of spitting. The use of the hooka is now much less, at least in upper India, than formerly; and it is an oriental luxury which is fast disappearing among the European officers, who find the cheroot by far the most convenient mode of enjoying the smoke of tobacco." P. 153.

All that we have to say, on the much-vexed point of tobacco-smoking, is contained in two simple prohibitions: don't spit—don't drink.

*Indication for the exhibition of Opium in Delirium Tremens, &c.*—There is, it must be confessed, a certain amount of practical truth in the following remarks; but the practitioner must be on his guard not to attach undue importance to the presence of any one symptom; his conduct should always be regulated by the general symptoms, and more especially by the state of the circulation—as ascertained by examining the action of the heart, as well as the arterial pulse.*

"It is difficult to say, what change takes place in the brain before sleep is induced, but a marked symptom of Delirium Tremens is a dilated state of the pupils; and according to Macnish the pupil during natural sleep is contracted. This dilatation of the pupils, though existing at the early period of Delirium Tremens, gradually diminishes under the use of laudanum, and other medicines, until it becomes unnaturally contracted as it often is in great excitement of the brain: in such instances, the contraction has evidently been brought about by the treatment, for if the disease runs its course to a fatal termination, the dilatation becomes more marked, until the pupil ceases to contract at all, even in the brightest light.

"The state of the pupil is a point of the utmost importance in the treatment of delirium, whether it be the effect of diminished or increased action in the brain; the dilated pupil being an index of the former state, and a contracted one of the latter: this has led to a successful practice by Doctor Graves of Dublin, who employs the combined effects of laudanum and tartar emetic, the former medicine being useful in delirium arising from a depressed state of the nervous system as in Horrors, and the latter from its sedative and relaxing effects, being equally efficacious in the delirium of excitement." P. 163.

---

* The following passage from Dr. Holland's Essay upon the subject, may be introduced with advantage here:

"The employment of opiates in cerebral affections is another question of much interest and various difficulty. It might be conjectured that there are some cases where the benefit is great; others, where injury alone can result; and experience fully confirms this presumption. Though it would be easy to detail particular cases in which one or other of these results is likely to ensue, I know no single principle, yet ascertained, by which they may be classed and distinguished. Perhaps one of the best practical tests might be found in the state of the pupil; known to be differently affected by opium, by belladonna, and certain other narcotics. Where contraction of the pupil is one of the symptoms of the disease, it may with some reason be supposed that opium is contra-indicated as a remedy; and a like inference might lead to the use of the opposite class of narcotics in its stead, transposing these methods, where the disease habitually produces a dilated state of the pupil. As far as my experience goes, it partially confirms the accuracy of the test, but is by no means sufficient to allow one to speak of it with certainty. We are still obliged to act here upon the suggestions of the time and symptoms present, or upon the experience which partial trials may afford."

In treating a case of decided *Delirium Tremens,* Dr. M. usually orders a drachm of Laudanum and one grain of emetic Tartar to be given every hour, until sleep is obtained; premising, in most cases, a turpentine enema. He advises also that the head be shaved, a blister applied on the crown, and the raw surface then dressed with a stimulating ointment. He does not approve of wine or any ardent spirits being allowed. When there is very great irritability of stomach, he has often found, he says, " the exhibition of five drops of Croton oil, either alone or combined with Opium, useful in restraining the nausea; if the stomach be loaded with bile (which it often is in such cases), free vomiting follows the use of the medicine, after which relief is experienced. After the delirious symptoms have entirely subsided, purgatives and tonics—such as the infusion of Chirayita, with diluted Sulphuric acid—will be necessary."

*Piles.*—This is a very common disease among soldiers, native as well as European, in the East Indies. Many cavalry and horse-artillery men are obliged to be transferred to the foot-service, in consequence of it; and not a few are disabled altogether by the supervention of Prolapsus of the rectum.

" Hemorrhoids and strictures of the urethra being common disorders are the means of removing many men from the mounted branch, though the latter affection is not so common a disease now as when injections were employed in the cure of Gonorrhœa." P. 172.

He points out the frequent connection of Piles, and also of hæmorrhage from the bowels higher up—this latter complaint is not unfrequent in India —with congestion of the Liver.

" In Hemorrhoids, as in many other diseases common to both temperate and tropical climates, the functions of the liver are often disordered, and this fact must be borne in mind in their treatment, whether they present themselves in the form of diarrhœa, dysentery, costiveness, obstruction or hemorrhoids; also, in a complaint which is sometimes met with in officers of long standing, I mean a flow of blood from the bowels; in the latter, the functions of the liver, and even the viscus itself may be disordered.

" Such a discharge is critical, and no doubt prevents a more serious illness; it must therefore be checked with caution." P. 173.

*Scurvy* is a disease of not unfrequent occurrence among the natives of India. It cannot, among them, be attributed to the use of salt meats or to a deficiency of vegetable food; neither can a cold damp climate be the producing cause, in their case. But the truth is that, whatever tends to enfeeble and deteriorate the general health, is apt, at certain times, to occasion symptoms of scorbutic cachexy. Hence—not to mention the influences of impure and vitiated air, in consequence of neglected ventilation in hospitals and prisons for example, and of an insufficiently nutritious diet, like that which the Hindoos live upon—these symptoms not unfrequently make their appearance after long-continued and repeated attacks of Fever and Rheumatism, after the injudicious use of mercury, and so forth. Upon this latter point, Dr. M. remarks that, " when calomel is accumulated in the system without producing salivation, and this at an unhealthy period of the year, it may undoubtedly give a tendency to the scorbutic disorder, and even produce some of its symptoms, such as pain in the shin-bones and disease of the gums." The following is the description of the disease, as it presents itself to the Indian practitioner.

"Land Scurvy, as affecting the native of India, is marked by a sponginess and purple appearance of the gums, and a leuco-phlegmatic or blanched countenance; pains in the shin bones which are often mistaken for Rheumatism, the integuments over these bones are puffy, and discoloured with livid spots resembling Echymoses: there is considerable debility which increases daily, and the digestion is greatly impaired. It is seldom, however, that all these symptoms are present at the same time, and hence the diagnosis is a matter of some difficulty when the rheumatic pains are merely present; when the gums become affected, the existence of the disease is easily recognised."* P. 177.

The only certain means of cure is change of air; the patient must be sent off to the hills without delay.

As in some degree allied to Scurvy, we may here introduce a few remarks on

*Dry Gangrene.*—This formidable affection is apt, as we have already stated, to be induced by the immoderate use of Calomel, especially when the drug does not produce its usual constitutional effects, nor excite ptyalism. The parts generally affected, under such circumstances, are the lips and cheeks. "There is a livid appearance around the mouth, or a circular patch of the same colour on the cheek. In severe Fevers which run their course in spite of every mode of treatment, and in which calomel has been exhibited to the utmost extent, this livid appearance continues until death, marking the existence of Gangrene which, no doubt, hastens the fatal termination."

The affected part of the face often becomes as black as charcoal. In many cases—more especially where symptoms of Dysentery had been present during life—there is ulceration and mortification of the intestines, at the same time. On the subject of treatment, the following practical remarks deserve the notice of the reader.

"As remedial means for Dry Gangrene when produced by calomel, those medicines used for the cure of mercurialization should be employed, such as the saline purgatives, nitric acid, sarsaparilla, and the frequent use of the warm-bath: the decoction of guaiacum, sassafras and the anuntamool or hemidesmus indicus are useful: the hydriodate of potass appears equally useful in removing the effects of mercury as in syphilitic cachexia, and may be used with advantage in Dry Gangrene when the effect of calomel.

"The free use of the warm-bath is perhaps, of all other remedial means, the most efficacious in removing the bad effects of mercury from the system; but in cases of Dry Gangrene, it can scarcely be employed to the requisite extent, as the patient is, for the most part, weak and debilitated. The nitro-muriatic

---

* The allusion in this passage to the rheumatic-like character of the pains in Scurvy suggests to our recollection that Sydenham has described a scorbutic species of Rheumatism. *Jam vero quantumcunque discriminis inter verum Rheumatismum et Scorbutum intercedat, aliam tamen speciem esse rheumatismi, quæ ad scorbutum quam proxime accedit, omnino confitendum est, utpote quæ insigniora hujus morbi symptomata æmulatur et pariter eadem ferme remedia sibi vendicat.*—Observ. Med. vi. 5.

bath may likewise be used, or rather the sponging, as a means of improving the general health, and the sulphate of quinine is to be administered, or the decoction of bark with diluted sulphuric acid.

"As local applications, the powder of bark is useful, so is the carrot poultice covered with camphor. In all gangrenous and sloughing sores, the carrot poultice is of the utmost importance.

"During the sickly season in the North-western Provinces, the application of blisters to native soldiers is often followed by sloughing sores, and the integuments, cellular and adipose substance are destroyed, laying bare the muscles which stand forth in bold relief; in such cases, the carrot poultice is the most efficacious in producing healthy granulations. In some of the worst cases of sloughing, the use of this vegetable as a poultice is almost miraculous." P. 187.

The chapter on *Rheumatism* is one of the least satisfactory of any in the book. The disease is common, both in its acute and in its chronic form, in India; and we are told that it is a frequent cause of disablement among the troops. It would seem, from what our author says, that it often prevails as an Epizootic, more especially among poultry; but what he means by saying that "it is not an uncommon occurrence to lose numbers of geese and turkeys from this disease, in the course of a single night," we cannot understand: rheumatism does not usually prove fatal so very rapidly, at least in the case of human beings. He seems to have very inaccurate notions respecting the connexion between acute Rheumatism and Cardial inflammation, nor does he even so much as allude to any of the auscultatory signs among the phenomena of the disease. His remarks, too, upon the treatment of it are exceedingly unsatisfactory. All that is said on the subject of acute Rheumatism is, that "the lancet is universally employed, and often with great success;" and, with respect to the chronic form, the information is most meagre and uninstructive, On the one hand, we read that the disease will sometimes yield to Peruvian bark, (ʒj. ter die); and, on the other, that "the use of calomel, so as to produce salivation, has often been found a cure for obstinate Rheumatism; and many medical officers employ this treatment exclusively; though effectual in curing the disease, it is doubtful whether a relapse in a worse form be not a common occurrence after the use of that drug."

Unless we have some therapeutic principle to guide us in the treatment of so diversified a malady as that which passes under the name of Chronic Rheumatism, our practice must always be empirical and wavering; there is, in truth, very nearly as much chance that we hit the wrong, as the right, nail upon the head. That many of the cases of the disease in the East Indies are attributable to the operation of a malarious agency, and partake largely of a *neuralgic* character, we have no doubt. It is in such cases that bark, quinine, and arsenic will prove most serviceable; but we cannot reasonably anticipate benefit from such medicines, as long as there is any febrile or inflammatory action in the system. One of the most important guides in our diagnosis of the true nature of each case, and consequently in its treatment, will be found to be the condition of the urinary secretion. But it is unnecessary to dwell upon this point here, as we expressed our views upon it, at some length, in the last number of this Journal, in the review of Dr. Robertson's work on Gout.

There are two or three other points in Dr. Macgregor's work which we had intended to notice, had our space permitted. What, however, has been said, and the extracts which have been given, will quite enable the reader to judge for himself of its merits and demerits. In spite of several obvious blemishes, it contains not a little of truly valuable information, and of very useful instruction. That subsequent experience has led the author to modify somewhat his over-exclusive views alike in theory and in practice, we have little doubt. A golden rule in the treatment of all diseases, mild or malignant, is—avoid extremes in the employment of any one remedy. Infinite mischief has been done by the neglect of this simple precept.

